# Versatile photonic molecule switch in multimode microresonators

**DOI:** 10.1038/s41377-024-01399-0

**Published:** 2024-02-20

**Authors:** Zihan Tao, Bitao Shen, Wencan Li, Luwen Xing, Haoyu Wang, Yichen Wu, Yuansheng Tao, Yan Zhou, Yandong He, Chao Peng, Haowen Shu, Xingjun Wang

**Affiliations:** 1 State Key Laboratory of Advanced Optical Communications System and Networks, School of Electronics, Peking University, Beijing, 100871 China; 2 College of Engineering, Peking University, Beijing, 100871 China; 3 School of Integrated Circuits, Peking University, 100871 Bejing, China; 4 Peking University Yangtze Delta Institute of Optoelectronics, Nantong, 226010 China; 5 Frontiers Science Center for Nano-optoelectronics, Peking University, Beijing, 100871 China; 6 Peng Cheng Laboratory, Shenzhen, 518055 China

**Keywords:** Integrated optics, Microwave photonics, Microresonators, Nonlinear optics

## Abstract

Harnessing optical supermode interaction to construct artificial photonic molecules has uncovered a series of fundamental optical phenomena analogous to atomic physics. Previously, the distinct energy levels and interactions in such two-level systems were provided by coupled microresonators. The reconfigurability is limited, as they often require delicate external field stimuli or mechanically altering the geometric factors. These highly specific approaches also limit potential applications. Here, we propose a versatile on-chip photonic molecule in a multimode microring, utilizing a flexible regulation methodology to dynamically control the existence and interaction strength of spatial modes. The transition between single/multi-mode states enables the “switched-off/on” functionality of the photonic molecule, supporting wider generalized applications scenarios. In particular, “switched-on” state shows flexible and multidimensional mode splitting control in aspects of both coupling strength and phase difference, equivalent to the a.c. and d.c. Stark effect. “Switched-off” state allows for perfect low-loss single-mode transition (Q_i_ ~ 10 million) under an ultra-compact bend size (FSR ~ 115 GHz) in a foundry-based silicon microring. It breaks the stereotyped image of the FSR-Q factor trade-off, enabling ultra-wideband and high-resolution millimeter-wave photonic operations. Our demonstration provides a flexible and portable solution for the integrated photonic molecule system, extending its research scope from fundamental physics to real-world applications such as nonlinear optical signal processing and sixth-generation wireless communication.

## Abstract (web summary)

The centrally located reciprocal point can achieve single-mode transmission and switch off the photonic molecule. The deviated reciprocal point can switch on the photonic molecule and dynamically control the splitting.

## Graphical abstract

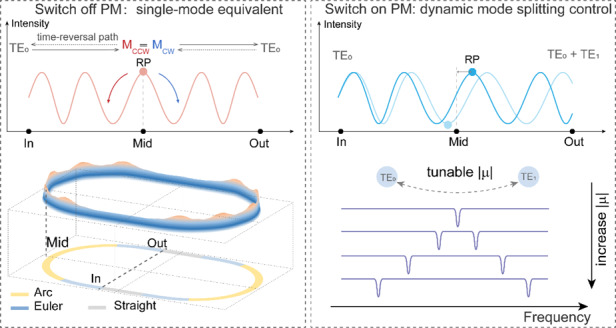

## Introduction

The interaction between optical modes results in the formation of hybridized supermodes and manifests as splitting in the spectrum, which is commonly referred to as photonic molecules (PMs)^1^. Such a physical system, analogous to two interacting atomic orbitals, exhibits discrete optical energy states and offers a diverse range of attractive properties^2–4^. In the realm of integrated photonics^5,6^, interaction within artificial PMs is typically induced through the coupling of resonant modes in joint microresonators^7^. Benefiting from precious manipulation of phases and energies, the microresonators-based PM is promised to assist frequency and phase matching in nonlinear optical processes, such as supporting Kerr comb generation^8,9^ and revealing various quantum optical phenomena like bound states in the continuum (BIC)^10^.

However, a key prerequisite for realizing the aforementioned applications is the ability to dynamically control the coupling of PMs. Despite extensive studies, manipulating the coupling strength still presents certain challenges. Utilizing the excitation of the time-varying electromagnetic/acoustic fields to effectively repulse the energy levels have been demonstrated^11,12^. But these complicated external driving approaches impeded the extensive adoption. Another method involves direct modulation of the coupling strength by mechanically changing the microresonators’ geometric factors^13–16^, which increases the complexity of fabrication processes. The microring resonator (MRR) can also be tailored by Bragg grating to enable coupling between clockwise (CW) and counter-clockwise (CCW) modes^17–20^ to achieve PMs, where the coupling strength is either fixed once fabricated, or relies on the modulated refractive index via high power induced nonlinear effect^19,20^.

Therefore, other formations of PM tuning mechanisms should be investigated to be more compact and flexible. The spatial-mode interaction, existing in the broadened-width waveguide, can also provide distinct resonances^21^. This type of waveguide is usually employed to reduce scattering loss and create MRR with higher intrinsic quality factor (Q_i_)^22,23^. Due to the absence of an effective method to regulate this interaction, a large bending radius was previously required to eliminate it for preventing degradation in Q_i_^23–25^. However, this operation hinders the observation of PMs, and also greatly reduces the free spectrum range (FSR), limiting the frequency tuning range in applications such as microwave photonics^26,27^.

In this study, we explored the regulatory mechanism of spatial-mode interaction in a broadened-width multimode racetrack-MRR (rMRR) to achieve PM. A modified coupled mode theory (CMT) allows for dynamically controlling the interaction between different spatial modes. As a result, the PM can transit between single and multi-mode working regimes, corresponding to “switched-off/on” functionality towards a wider application range. The “switched-off” PM prevents the higher-order spatial mode from spreading throughout the resonator, enabling high-Q single-mode transmission without limitations on the bending radius. Following this design, we have experimentally demonstrated a foundry-based silicon microring with the Q_i_ of nearly 10 million (0.96 × 10^7^) and the FSR of up to 115 GHz. This displays the highest product of Q_i_ × FSR on the silicon-on-insulator (SOI) platform to date. Based on it, we have successfully verified an ultra-wide tunable integrated millimeter-wave photonic (IMMWP) filter, operating at frequencies up to 57.5 GHz (U band), with an exceptionally narrow 3-dB bandwidth of 32 MHz. Moreover, an integrated optoelectronic oscillator (OEO) has been achieved, exhibiting a tunable frequency range of 50 GHz (the first OEO based on the MRR with such a high-frequency range). Meanwhile, the “switched-on” PM can be flexibly tuned to generate the higher-order spatial mode throughout the rMRR. Both the coupling strength and phase difference of the supermodes can be adjusted. In this case, we demonstrated that the frequency interval of the supermode splitting can be dynamically tuned within a range exceeding 5.7 GHz. This work offers a fresh perspective on device design and energy manipulation of integrated microresonators, making a significant step toward next-generation ultra-high-speed optoelectronic applications. Simultaneously, it sheds new light on the physical aspects of quantum and nonlinear optics.

## Results

### The regulatory mechanism of spatial-mode interaction in bending structure

**Fig. 1:**
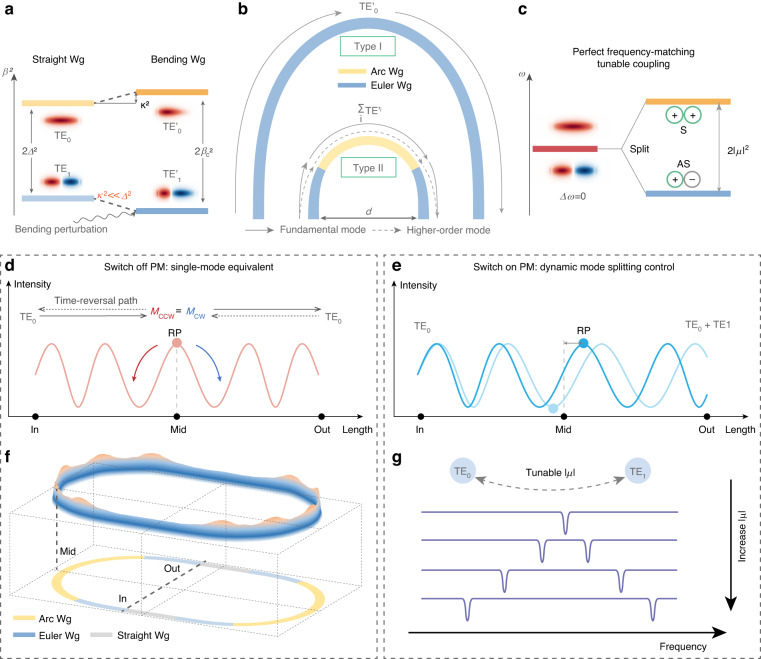
**Concept of the mechanism of regulating the spatial-mode interaction and controlling the PM**. **a** The level diagram of the propagation constant with (right)/without (left) bending perturbation, along with the relationship between *κ*, *Δ* and *β*_*c*_; **b** Two different types of the 180° bending waveguide. Type I: Euler bend with a large chord length. Only TE_0_ mode exists throughout the bend. Type II: the combination of Euler bend and arc bend with a small chord length. Higher order mode also exists throughout the bend; **c** The frequency level diagram represents the resonances from different spatial modes in the same frequency, analogous to a split energy level in a diatomic molecule. S: symmetry; AS: anti-symmetry **d** The centrally located reciprocal point can achieve equivalent single-mode transmission and “switch off” the photonic molecule. **e** The deviation from the center of the reciprocal point can determine the coupling strength between spatial modes. Dark blue and light blue represent different coupling situations. **f** The schematic diagram of energy distribution in the entire MRR of the “switched-off” PM. **g** The illustration of the tunable mode splitting of the “switched-on” PM. *κ*: coupling ratio between spatial modes; *Δ* and *β*_*c*_: The difference in the propagation constants of the two modes in the straight and bending waveguide, respectively; TE_i_: transverse electric mode in the straight waveguide; TE’_i_: transverse electric mode in bent waveguide; Wg: waveguide; d: the chord length of the bent structure; M_CCW_: The field evolution process from “Mid” to “In”, which is the time-reversal path; M_CW_: The field evolution process from “Mid” to “Out”; RP: reciprocal point; PM: photonic molecule; *μ*: coupling rate between two modes

Figure 1a illustrates the shifts in propagation constants for both the fundamental mode and higher-order mode within a width-broadened waveguide, before and after undergoing bending perturbation. In a straight waveguide, the transverse electronic (TE_n_) modes exhibit orthogonality without any inter-mode coupling and the difference in the propagation constants of the two spatial modes can be denoted as Δ. This characteristic can also be approximated in a curvature constant bending strip waveguide on isotropic materials like Si or silicon nitride (SiN). When the curvature is varied, it can be viewed as a perturbation that opens up the coupling channel between these spatial modes. At this time, the difference in the propagation constants for the modes within the bent structure is written as *β*_*c*_. According to the coupling mode theory, the correlation between *Δ* and *β*_*c*_ can be written as follows^28^:

1 $${\beta }_{c}^{2}={\varDelta }^{2}+{\kappa }^{2}$$

where *κ* represents the power coupling ratio between different spatial modes. Considering a low field overlap between two spatial modes, *κ* should be a weak value compared with *Δ*. Thus an approximation of *κ*^2^ ≪ *Δ*^2^ is applied, which is the well-developed rotating wave approximation (RWA) for Stark shift effect in atomic physics^29^. In this case, the analytical solution of the coupled mode equations should be reformulated. A detailed derivation process is provided in Fig. S1 in Supplementary Note I. Here, we present the electrical field distribution of the TE_0_ and TE_1_ in two bending structures, Euler bend (EB) and arc bend. Euler bend has a continuous variation in curvature while the arc bend maintains a constant curvature. When only TE_0_ is the input stimulus as the boundary condition, the field distribution can be written as:

2 $${E}_{0}\left(z\right)={{\rm{C}}}_{0}{e}^{-j{\beta }_{0}\left(z\right)z}$$

3 $${{\rm{E}}}_{1}\left({\rm{z}}\right)=\left\{\begin{array}{ll}\frac{\kappa }{{\beta }_{c}}\sin \left({\beta }_{c}z\right){e}^{-j\frac{\left({\beta }_{1}\left(z\right)+{\beta }_{0}\left(z\right)\right)}{2}z-j\frac{\pi }{2}}, & {\text{in}}\; {\text{EB}}\\ {{\rm{C}}}_{1}{e}^{-j{\beta }_{1}\left(z\right)z}, & {\text{in}}\;{\text{arc}}\end{array}\right.$$

Where *E*_0_ (*E*_1_) represents the field of TE_0_ (TE_1_). *β*_0_ (*z*) and *β*_1_ (*z*) represent the propagation constant of TE_0_ and TE_1_, respectively. C_0_ and C_1_ represent the constants of amplitude.

### The principle of dynamically controlling the PM based on spatial-mode interaction

We designed a rMRR to serve as the platform for the dynamic tunable PM. In this configuration, the 180° bending waveguide is designed as a Type II structure, as shown in Fig. 1b. This structure is a combination of Euler bend and arc bend. Compared with the Type I waveguide which is wholly composed of Euler bends with a larger chord length, Type II structure can stimulate TE_1_ inside the bend when TE_0_ is injected into the bend due to its relatively larger curvature change rate. Therefore, both TE_0_ mode and TE_1_ mode can form resonance modes, providing two discrete energy states for the PM, as depicted in Fig. 1c

Moreover, since the bending waveguide contains two spatial modes with different propagation constants (*β*_0_ (*z*) and *β*_1_ (*z*)), it results in an intensity distribution pattern featuring alternating phases of coherent constructive and destructive interference (as depicted in Fig. 1d, e). According to the conclusion of the last section, we can calculate the intensity distribution in the bending structure according to Eqs. (2) and (3):

4 $$I\propto \left({E}_{1}+{E}_{0}\right)\cdot \left({E}_{1}^{* }+{E}_{0}^{* }\right)$$

where *I* represents the spatial intensity interference pattern inside the bending waveguide and the symbol * represents the complex conjugate. Then, we introduce the concept of the reciprocal point, defined as the intensity extreme point near the center of the bend. Using Eq. (4), the position of the reciprocal point can be easily obtained without the calculation of *κ*, which greatly simplifies the computational complexity (See Supplementary Note I for a detailed explanation).

Then, we will explain how to control the switching state of PM by manipulating the position of the reciprocal point. Given the high symmetry of the bending structure, when TE_0_ is injected at the input port (Labeled “In” in Fig. 1d, f) with the reciprocal point located at the middle (labeled “Mid”), the intensity distribution pattern is symmetrical along the propagation direction of the waveguide, as depicted in Fig. 1d. In this case, only TE_0_ can be found at the output port, which is the same as the input port, because the time reversal power flow from “Mid” to “In”(Labeled M_CCW_) is equal to M_CW_ (strict formula proof is available in Supplementary Note II). This results in the perfect equivalent single-mode transition. Since TE_1_ cannot resonate in rMRR, the system now has only one energy level, thus rendering the PM “switched off”. An overview of the intensity distribution across the entire rMRR is presented in Fig. 1f. The bending waveguide exhibits an interference pattern, while the straight waveguide maintains a uniform distribution.

Subsequently, if the parameters are adjusted in Eq. (4) to make the position of the reciprocal point deviate from the center, the TE_1_ mode will be remained at the output, as shown in Fig. 1e. It results in the dual-mode resonances in the cavity, where the coupling strength determines the distance of two energy levels. The PM is “switched on” now. The offset of the reciprocal point becomes a reference to determine the coupling coefficient. This enables tunability in the strength of mode splitting, realizing the dynamic control of the PM (Fig. 1g). Notably, the local modification of the refractive index in the bent region, simply through the thermo-optic effect, allows for the realization of this dynamic control.

### Simulation, fabrication and basic characterization of the rMRR-based PM

**Fig. 2:**
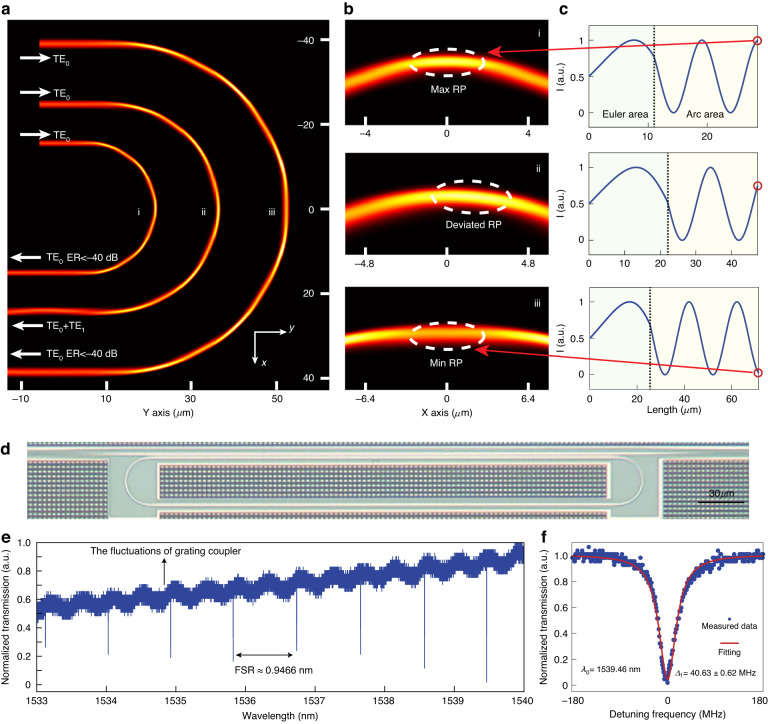
**The design and characteristics of the controllable rMRR-based integrated photonic molecule. a** Three-dimensional electromagnetic power flows obtained via finite-difference time-domain (FDTD) simulation for different bend structures. The straight waveguide’s width increases from 2 μm to 3 μm from inside to outside, respectively. **b** Enlarged images display the interference pattern in the middle of the bends with **i** maximal RP, **ii** deviated RP and **iii** minimum RP situations, respectively. **c** Calculation results of half-bending length using the modified CMT under RWA, which are consistent with the electromagnetic power flows in (**a**, **b**). The x-axis presents the path length along the centerline of the bending structure. **d** Optical microscope image of the fabricated rMRR. **e** Measured spectral responses at the through port of the fabricated silicon high-Q rMRR with an ultra-large FSR. **f** Measured resonance peaks (blue points) at different resonance wavelengths. The red line shows the Lorentzian transmission matrix model fitted. RP: reciprocal point

We employed the silicon photonics platform to validate the efficacy of the proposed method. The strip waveguide possesses the standard thickness of 220 nm, covered with SiO_2_ cladding. The operating wavelength is set at 1550 nm. Using Eqs. (2–4), the position of the reciprocal point can be manipulated by adjusting the length ratio of the Euler bend and arc bend with continuous curvature at their connections. The width of the waveguide is also continuously and linearly changed with the curvature to introduce more design degrees of freedom. As the structure is symmetrical, it suffices to calculate the interference patterns for only half length of the bent waveguide. We proposed three bent waveguides with chord lengths d of 30 μm, 48 μm, and 76 μm as different coupling situations. The widths of the input straight waveguide are 2 μm, 2.5 μm, and 3 μm, respectively (The details of the structure can be seen in Fig. S2 in Supplementary Note III). The three-dimension finite difference time domain (FDTD) method was performed to calculate the intensity distribution in bent waveguides, as shown in Fig. 2a. The intensity distribution of the proposed bent waveguides shows distinct constructive and destructive interference patterns. The enlarged views of the center are shown in Fig. 2bi-iii. Figure 2bi, iii show the cases where the max and min reciprocal points are located exactly in the center. The corresponding bent waveguides feature a good single-mode transmission, with the mode extinction ratio (MER) ≤ −40 dB (defined as $10{\log }_{10}\left({T}_{{\rm{T}}{{\rm{E}}}_{1}}/{T}_{{\rm{T}}{{\rm{E}}}_{0}}\right)$). The transmission of TE_0_/TE_1_ in bending structure Fig. 2ai is −0.0005 dB/-59.6170 dB @ 1541 nm (define as $10{\log }_{10}{T}_{{\rm{T}}{{\rm{E}}}_{0}{\rm{or\; T}}{{\rm{E}}}_{1}}$, where *T* represents normalized power transmission).The simulation results indicate that the proposed structure can support the optical bandwidth greater than 24 nm while maintaining the MER ≤ −30 dB. When the waveguide width fluctuates ±10 nm, the optical bandwidth is still wider than 20 nm (Details can be seen Figs. S3 and S4 in Supplementary Note III). For the case where the reciprocal point is offset from the center in Fig. 2bii, the field at the output port shows the mixture of TE_0_ and TE_1_. Figure 2c shows the calculated pattern from Eqs. (2–4) (Details of the calculation process and the results can be seen in Fig. S5 in Supplementary Note IV). The results have good agreement with Fig. 2b, c, where the positions of the reciprocal points are well matched. These results reveal that the modified CMT under RWA works well for manipulating the spatial-mode interaction.

It is important to emphasize that even within the same bend structure, the thermo-optic effect can be used to change the refractive index of the material, thereby modifying the distribution of interference patterns along the bent waveguide. In Fig. S6 in Supplementary Note V, we conducted additional numerical simulation calculations to explore the principle of using the thermal-optics effect to manipulate the position of reciprocal point, as well as its relationship with the power conversion between modes at the output port.

The i bent waveguide in Fig. 2a, which possesses an impressively reduced effective radius of merely 18.2 μm, is utilized in the construction of the rMRR with the straight waveguide length of 290 μm. The proposed device was fabricated on a standard 220 nm silicon on insulator (SOI) platform. Figure 2e presents the measured spectral response at the through port of the rMRR. One of the significant advantages of the “switched-off” PM rMRR is the ultra-large FSR (115 GHz), which is achieved through the ultra-compact bend without relying on an exceedingly large footprint to suppress multimode generation throughout the rMRRs. Figure 2f shows the enlarged view of the resonance peaks. The resonance at 1539.46 nm owns the mean full width at half maximum (FWHM) of 40.63 ± 0.62 MHz with critical coupling, indicating that the loaded Q (Q_l_) = 0.48 × 10^7^ and Q_i_ = 0.96 × 10^7^, nearly 10 million^30,31^. The mode splitting caused by the backscattering is also observed, which is common in high Q resonance cavities^32^ (These resonances can be seen in Fig. S7 Supplementary Note VI). This innovative manipulation to switch off the PMs greatly enhances the performance of MRRs (Q, FSR and footprint), representing a transformative breakthrough that has not been previously considered. This approach holds promise for advancing other fields, such as wide-range RF operation, as demonstrated in the following section.

### The “switched-off” photonic molecule: towards ultra-wideband and high-resolution RF operation

**Fig. 3:**
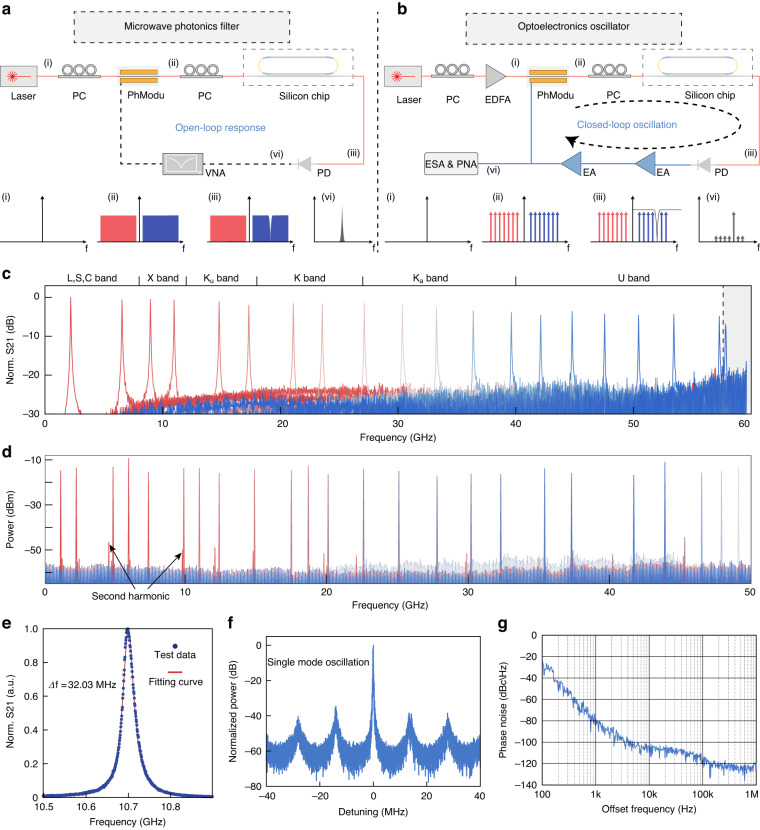
**The demonstration of the ultra-wideband tunable integrated RF filter and integrated OEO based on the “switched-off” photonic molecule rMRR**. **a** The schematic of the structure of the integrated RF filter. Using different colors (red and blue) to represent π phase difference between two sidebands. **b** The schematic of the structure of the integrated optoelectronic oscillator. **c** The measured normalized S21 transmission response multi-frequency band-pass filter from sub-6G band to U band with a under coupling state resonance. Different colors represent measurement results from different trials at various frequencies. The gray area represents the frequency range beyond half of the FSR and is considered to be the invalid frequency range here. **d** The measured RF spectrum of the microwave signal generated by the wide tunable range OEO with the resolution bandwidth (RBW) of 1 MHz. Different colors represent measurement results from different trials at various frequencies. **e** Zoom-in view of the S21 response from (**c**). The 3-dB bandwidth is 32 MHz. **f** Spectrum with a span of 80 MHz with the RBW of 100 kHz. The side modes are fully suppressed with 38 dB attenuation, implying that the filter owns a good frequency selectivity. **g** The phase noise characteristic of the generated signal. PC polarization controller, PhModu phase modulator, PD photodetector, VNA vector network analyzer, EA electric amplifier, ESA electrical spectrum analysis, PNA phase noise analyzer

In some applications such as integrated microwave photonics^33^, the FSR of the MRRs determined the valid operation frequency range of the system^26,34^. Although multifunctional filters are achieved with reconfigurable circuits, the frequency tuning ranges are narrow, mostly below 20 GHz^35^, limited by the FSR of MRRs. Therefore, we used the “switched-off” PM rMRR to implement two ultra-wideband and high-resolution microwave and millimeter-wave applications: an IMMWP filter and an OEO. The illustration of the experimental setup of the filter is shown in Fig. 3a. An external laser was used to generate continuous wave light into the phase modulator (PhModu) via a polarization controller. Then the other PC was connected to send the light to the silicon chip. Finally, the light was collected by a photodetector (PD). A vector network analyzer was used to analyze the S21 response of the system. The experiment details can be seen in “Material and methods” section. Using the phase-modulation to intensity-modulation conversion scheme^36^, a tunable band-pass filter with a frequency range of half of the FSR was achieved, as shown in the measured normalized RF transmission in Fig. 3c. The wide tunable range of over 57.5 GHz was successfully achieved (from L to U band), which is the result of inheriting the high performance of the rMRR. The enlarged image in Fig. 3e reveals the single passband of the proposed filter, with a Lorentzian fit curve (red line) indicating a 3-dB bandwidth of 32 MHz. The details of the filter’s passbands at other frequency bands can be seen in Fig. S8 in Supplementary note VII. Remarkably, the proposed IMMWP filter represents the widest tunable range achievable with an integrated device on a silicon platform, while still maintaining a narrow 3-dB bandwidth. On this basis, we further realized the wide-range tunable rMRR-based OEO and the experimental setup is illustrated in Fig. 3b (The setup details can be seen in “Material and methods” section). Figure 3d shows the measured RF spectrum by an electronic spectrum analyzer (ESA) with a range of 50 GHz. The higher frequency range is sheltered by the detection frequency range of the ESA and limited by the bandwidth of the PD. The enlarged image of the spectrum is shown in Fig. 3f. The side-mode oscillation can be clearly observed with a 14 MHz frequency interval from the main mode. The side modes are fully suppressed with nearly 38 dB attenuation, indicating that the optical response of the high-Q MRR is well mapped into the RF domain and the side modes are filtered effectively. Figure 3g shows the measured phase noise of the generated signal at around 12 GHz. The phase noise at 10 kHz offset frequency is measured as −105 dBc/Hz. The implementation of wideband and high-resolution RF signal processing and generation represents a significant advancement in the field, with promising implications for the development of six-generation (6 G) wireless communication technologies to provide ultra-wideband operation.

### The “switched-on” photonic molecule: dynamic mode splitting manipulation

**Fig. 4:**
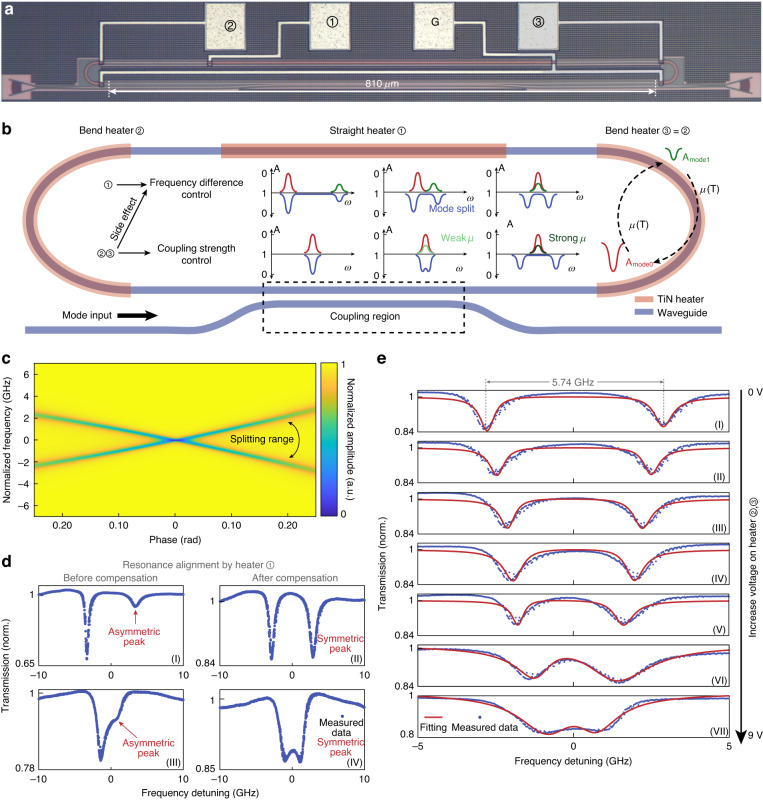
**Dynamic mode splitting control in the “switched-on” photonic molecule. a** The optical image for the fabricated rMRR. **b** The operation schematic of the splitting control via different spatial modes in a single rMRR. **c** the relationship between the phase difference of two spatial modes when they are in the middle and the spectral bifurcation physical properties. **d** The function demonstration of heater ①: the compensation of frequency deviation between two resonances for consistent extinction ratio and symmetric splitting peak. **e** The demonstration of the dynamic strength control of the splitting. The controllable frequency shifts up to 5.74 GHz (~0.12 FSR). G: grounded wire

Then, we demonstrated the dynamic control of the PM by effectively changing the spatial-mode coupling in the rMRR. First, we employed numerical calculations to further investigate the correlation between the coupling strength and the spectral response of the cavity, which allows extensive investigation into the PMs behavior regarding the spectral bifurcation properties. We assess the relative phase difference ∆*φ* between the TE_0_ and TE_1_ at the center of the waveguide to determine the position of the reciprocal point. The calculated transmission spectra are depicted in Fig. 4c and show obvious bifurcation physical properties with the change of ∆*φ*. When ∆φ = 0, there is no mode splitting, corresponding to the minimal coupling rate. As the phase difference continues to increase, the frequency bifurcation physical properties emerge. The large mutual coupling rate corresponds to the higher frequency doublet gap. Thus, the mode coupling induced spectral splitting could be tuned. (More details can be found in Figs. S9 and S10 in Supplementary Note VIII.)

Then, we utilize the thermo-optics effect to experimentally achieve the dynamic control. The splitting spectrum (*ω*^(s,as)^) for the rMRR can be described as^8^:

5 $${\omega }^{\left({\rm{s}},{\rm{as}}\right)}=\frac{\left[{\omega }_{m}+{\omega }_{n}\right]}{2}\pm {\left\{\frac{{\left[{\omega }_{m}-{\omega }_{n}\right]}^{2}}{4}+{\left|\mu \right|}^{2}\right\}}^{\frac{1}{2}}$$

where *μ* is the coupling rate between the two modes. *ω*_*m*_ and *ω*_*n*_ are the resonant frequencies of the spatial modes in the cavity. As shown in Fig. 4a, for electronically dynamic control, the TiN micro-heaters were applied on the bent waveguides (labeled ②, ③) and straight waveguide (labeled ①), respectively. With the same bend dimension, the straight waveguide is intentionally elongated as 810 μm and the length of the microheater ① is 500 μm to increase the modulation efficiency of the thermo-optics effect. The operation principle is depicted in Fig. 4b. Due to the different responses of propagation constants of TE_0_ and TE_1_ to the applied thermal field, the phase difference between the two modes will be tuned as the applied voltage. At the straight waveguide region, there is no inter-mode coupling, and the applied voltage on Heater ① will change the frequency difference between the two modes, as $\varDelta \omega ={\omega }_{m}-{\omega }_{n}$. At the bent region, the applied voltage on Heater ② and ③ will offset the position of the reciprocal point, leading to a tunable *μ*. It also has the side effect to change the frequency difference, same function as Heater ①. Under the same *μ*, the splitting will be the strongest when the *Δω* is 0. In the experiment, the voltage applied on Heater ② and ③ is changed to tune the coupling coefficient and the voltage applied on Heater ① is tuned to compensate for the change of *Δω* caused by Heater ② and ③.

For the demonstration of dynamically controlling the PM, the spectral range is set as around 1600 nm, which is far away from the effective range with low MER. When no voltage is applied to all heaters, the measured spectrum of the rMRR is shown in the upper panel of Fig. 4d.I. The target mode splitting has two asymmetric peaks. After compensating via applying voltage on heater ①, the two peaks have the same depth, marking a near-zero *Δω*, as shown in Fig. 4d.II. Figure 4d.III, IV exhibit the same process when the voltage on Heater ②, ③ is 9.5 V. Next, to display that we can freely control the strength of the mode splitting, we then sequentially applied the increasing voltage on Heater ②, ③ from 0 V to 9.5 V, which is the maximum voltage that the TiN heaters can be endured. We maintain an equal voltage (V_2_ = V_3_) on both heaters throughout the entirety of the experiment and data recording process. Meanwhile, we changed the voltage applied on Heater ① every time before recording the spectrum to ensure $\varDelta \omega =0$, so that the frequency drift caused by the side effect of micro heater ②, ③ could be compensated and the mode splitting is symmetric. As shown in Fig. 4e, the strength of mode splitting can be controlled from 5.74 GHz to 1.76 GHz, which is a concise way to achieve dynamic control of the PM. The extinction ratio of the measured spectrum is not high enough due to the additional unexpected propagation loss caused by the micro-heater. This can be improved by redesigning the coupling region and increasing the coupling coefficient between the bus waveguide and the rMRR.

## Discussion

**Fig. 5:**
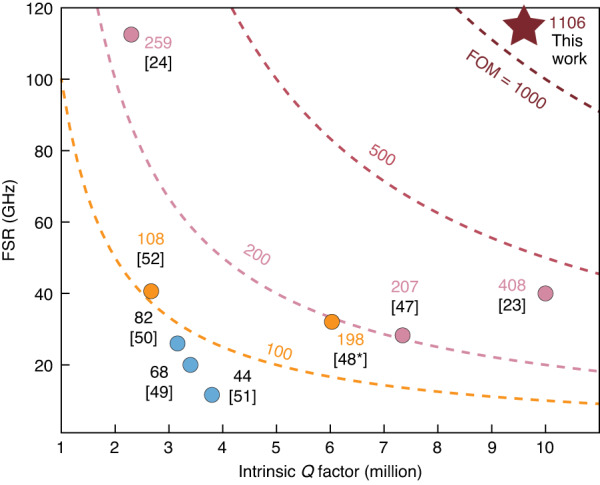
**Summary of the performance of the microring in silicon platform**^23,24,47–52^. ^*^ Considered as critical coupling

To benchmark the performance of the dynamic PM based on the spatial-mode interaction, we make a comparison of the existing works, summarized in Table 1. The spatial-mode interaction approach proposed here enables the dynamic control of PMs in a simple manner with a wide tuning range of 0.124 FSR. A wider splitting dynamic range would be observed if the micro-heater could withstand higher voltages. In the case where the PM is “switched off” in the proposed rMRR, Fig. 5 summarizes the performance comparison with existing high-Q MRRs based on SOI platform. As expected, all of these previous works are limited by the compromise between Q factor and FSR, resulting in a choice between achieving either ultra-high Q or ultra-large FSR. To fully demonstrate the superiority, we define a figure of merit (FOM) as ${\rm{FOM}}={{\rm{Q}}}_{{\rm{i}}}\times {\rm{FSR}}$, which is similar to the definition of finesse^37^. As illustrated, here we exhibit the largest FOM among the compared work. Especially, our work is based on standard process without any specialized low-loss fabrication optimization, and thus could be seamlessly utilized in large-scale integrated systems.

**Table 1:** Comparation of different schemes of dynamic photonic molecule

	Number of microcavity	Dynamic control method	Splitting shift range / FSR
Zhang^11^	Dual	amplitude of microwave	~ 4 GHz/~ 2 nm
Kapfinger^12^	Dual	amplitude of acousto	~ 0.5 meV/-
Sato^45^	Dual	optical pulse	-
Siegle^13^	Dual	mechanism(ES*)	-
Woska^14^	Dual	mechanism(ES)	520 pm/-
Yang^16^	Triple	mechanism(PZT**)	~ 1.5 GHz/-
Wang^15^	Triple	mechanism(PZT)	5 GHz/-
Lu^8^	Single	-***	~ 0.8 nm/~ 7.192 nm
Xu^46^	Dual	-***	~ 2.3 nm/~ 3.4 nm
(This work)	Single	spatial-mode interaction	5.74 GHz/46.38 GHz

*ES elastomer substrate,

**PZT Lead Zirconate Titanate, ***The photonic molecule is fixed once fabricated

It should be noted that the proposed approach ensures an ultra-large FSR for the “switched-off” PM with a 24 nm optical bandwidth of low MER. However, for high-speed applications such as IMMWP, where a wider FSR is essential to accommodate the wideband signals, which is more significant rather than a higher optical bandwidth in systems with single optical carrier. Therefore, the “switched-off” PM rMRR turns out to be an excellent match and has the potential to revolutionize these fields. The performance of the RF application could be further improved by architecture optimization. For wide operation range, with intensity modulation methods to construct the IMMWP filter can explore the entire FSR. Setting the rMRR as the under-coupled state could achieve a narrower filter bandwidth. Our rMRR offers the unprecedented capability to continuously tune the frequency over an ultra-wideband, which is urgently required for the 6G communication to push the operation bandwidth towards terahertz band and solve the problem of scarce spectrum sources. And incorporating the proposed microring with its switch-off state into the microwave photonics architecture^26^ holds the promising potential to achieve an all-spectrum reconfigurable front-end for 6G application^38^. A series of applications including reconfigurable signal process^39^, carry-generation for wireless transmission^40^ and tunable local oscillator^41^ for down-converting various received signals can be expanded from the sub-6G band to U band, leading into the field of integrated millimeter-wave photonics.

Our approach is highly platform-portable. It can be migrated to other material platforms like SiN^42–44^, and Lithium niobate^30^ to manipulate the spatial-mode interaction in those width-broadened waveguides, where it is expected to generate new physical mechanisms with dynamic PM. Also, in Figs. S11 and S12 in Supplementary Note IX, we simulated the proposed bend for other material platforms such as SiN to show the universality of high-Q design. Moreover, since we propose a modified coupled-mode theory in the bent waveguide, the computational complexity is greatly simplified. The simplified calculation method is conducive to the use of artificial intelligence algorithms to achieve inverse design, so as to achieve better performance, in single-mode transmission or mode splitting control, etc, shedding light on novel avenues for exploration and application, from fundamental physics to practical applications scenarios.

## Materials and methods

### Experiment setup for Q factor measurement

The Q factor measurement is performed by scanning an external-cavity-diode laser (Toptica CTL 1550) across a resonance from the blue side to the red side. To avoid the distortion of line shape caused by thermal nonlinearity, the on-chip power is reduced to ~ −30 dBm. The frequency of the laser is referenced to a Mach-Zehnder interferometer, which is calibrated by an optical time domain reflectometer. The optical signal is collected by an avalanche photodiode and recorded by an oscilloscope (Keysight MXR404A). The data was repeated 15 times and averaged.

### Experiment setup for IMMWP filter and OEO

To achieve the rMRR-based IMMWP filter, an external laser (Toptica CTL 1550) was used to generate continuous wave light with the power of ~15 dBm into the phase modulator (PhModu) via a polarization controller (PC) to correct the polarization of the light. The high-speed commercially available PhModu (EOspace PM-DV5-60-PFA-PFA-SPK722) with E-O bandwidth of 60 GHz modulated the microwave and millimeter wave into the optical domain and generated two balanced optical sidebands with a π phase difference with respect to the optical carrier. The polarization of the light after PhModu was corrected again by PC2. Then it was launched into the silicon chip through a grating coupler with ~6 dB insertion loss. The proposed rMRR was used to tailor the modulated sideband so that the optical response of the rMRR would be consistently transferred to the RF domain. The signal was finally collected by a photodetector (PD, Finisar XPDV2120R-VF-FP) and the recovered RF signal was characterized by a vector network analyzer (VNA, Keysight N5247A). For the data presented in Fig. 3c, we configured the frequency scan range of the VNA to span 60 GHz. It is important to note that within the 0 to 57.5 GHz range, a single passband is observed, attributable to the ultra-wide FSR of the microring. By adjusting the central wavelength of the laser, the center frequency of the filter passband can be changed accordingly. The color gradient in Fig. 3c, transitioning from red to blue as it moves from the lower to a higher frequency band, illustrates the progressive shift in the center frequency of the filter passband. The experimental testing of the OEO is based on the setup of the filter, with a distinct procedure followed after the PD. The recovered RF signal from PD was amplified by the electric amplifier (EA, SHF 807 C). A 3-dB RF power splitter (not shown in the figure) was used to equally divide the output signal. In one path, the signal was feedbacked to the PhModu and an optoelectrical hybrid loop was formed. In the other path, the signal was launched to an electrical spectrum analyzer (ESA, Keysight 9010B) and a signal source analyzer (Keysight E5052B) for frequency range and phase noise characterization. In Fig. 3d, the resolution bandwidth (RBW) of the ESA is configured as 1 MHz, and the video bandwidth (VBW) is configured as 200 kHz. In Fig. 3f, the resolution bandwidth (RBW) of the ESA is configured as 100 kHz, and the Video Bandwidth (VBW) is configured as 51 kHz.

### Supplementary information

Supplementary information for Versatile Photonic Molecule Switch in Multimode Microresonators

## Data Availability

The data that supports the plots within this paper and other findings of this study are available from the corresponding authors upon reasonable request.

## Code availability

The codes that support the findings of this study are available from the corresponding authors upon reasonable request.
