# Supplementary material for: Versatile photonic molecule switch in multimode microresonators
Source: Light Sci Appl. 2024 Feb 20;13:51. doi: 10.1038/s41377-024-01399-0 (PMC10876944; doi:10.1038/s41377-024-01399-0)
Supplement: Supplementary file 1 — Supplementary information for Versatile Photonic Molecule Switch in Multimode Microresonators [file 41377_2024_1399_MOESM1_ESM.pdf]

Supplementary Information for

Versatile Photonic Molecule Switch in Multimode Microresonators

Zihan Tao[1], Bitao Shen[1], Wencan Li[1], Luwen Xing[2], Haoyu Wang[3], Yichen Wu[1], Yuansheng Tao[1], Yan Zhou[4], Yandong He[3], Chao Peng[1,5,6], Haowen Shu[1,*], Xingjun Wang[1,4,5,6,*]

[1]State Key Laboratory of Advanced Optical Communications System and Networks, School of Electronics, Peking University, Beijing, 100871, China.

[2]College of Engineering, Peking University, Beijing, 100871 China.

[3]School of integrated Circuits,Peking University, 100871 Bejing, China

[4]Peking University Yangtze Delta Institute of Optoelectronics, Nantong 226010, China.

[5]Frontiers Science Center for Nano-optoelectronics, Peking University, Beijing 100871, China.

[6]Peng Cheng Laboratory, Shenzhen 518055, China.

Corresponding authors: *haowenshu@pku.edu.cn, *xjwang@pku.edu.cn.

Supplementary note I: Details about the modified coupled mode equations

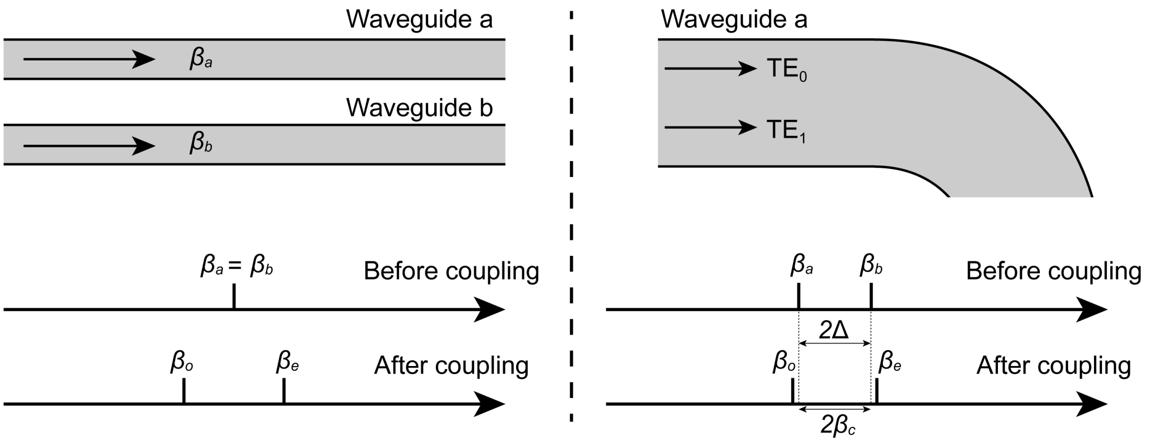

Fig. S1: The comparison between different coupling situations. Left: the traditional case with the same propagation constants. Right: the coupling case with different constants.

As shown in Fig.S1, we make a comparison of the two different coupling situations. The left one represents the conventional coupling case, where two waveguides are closed to each other and the power is exchanged between them. Usually, we deal with the case that the width of the two waveguides is the same so that the propagation constant difference Δ is zero. However, the coupling process between different spatial modes is far from the typical case, since the propagation constants are different due to the different mode properties. So we established the appropriate modified coupled mode equations to quantitatively describe the physical phenomenon in the proposed bend and prove the feasibility of this approach. This model can also achieve a concise design method without laborious numerical calculations of parameter sweeps. The field in the proposed bend can be expressed using the following equations:

$$E_0(z) = A(z) * e^{-j\beta_0(z)z} \tag{1}$$

$$E_1(z) = B(z) * e^{-j\beta_1(z)z} \tag{2}$$

$$\frac{\mathrm{d}A(z)}{\mathrm{d}z} = -j\kappa(z)B(z)e^{-j(\beta_1(z)-\beta_0(z))z} \tag{3}$$

$$\frac{\mathrm{d}B(z)}{\mathrm{d}z} = -j\kappa(z)A(z)e^{+j(\beta_1(z)-\beta_0(z))z} \tag{4}$$

where $E_0(E_1)$ represents the field of TE_0 (TE_1). $A(z)$ and $B(z)$ are the amplitude of TE_0 and TE_1 mode propagating in the bent waveguide. $\beta_0(z)$ and $\beta_1(z)$ represent the propagation

constants of the two modes. $\kappa(z)$ is the coupling coefficient between the two modes. z represents the propagation along the bent waveguide. Assumed that there is only $\mathrm{TE_0}$ mode launched into the bent waveguide, so the boundary condition to solve the above equation is $A(0) = 1$, $B(0) = 0$. Then the $A(z)$ and $B(z)$ can be summarized as follow:

$$A(z) = e^{-j\Delta z}\left(\cos\beta_c z + j\frac{\Delta}{\beta_c}\sin\beta_c z\right) \tag{5}$$

$$B(z) = e^{+j\Delta z}\frac{-j\kappa}{\beta_c}\sin\beta_c z \tag{6}$$

where $\Delta = \frac{(\beta_1-\beta_0)}{2}$ represents the propagation constant difference between two modes. $\beta_c = \sqrt{\kappa^2+\Delta^2}$ represents the propagation constants difference after being disturbed in the bent waveguide (where the inter-mode coupling occurs). Since the electric field distributions of the two modes are quite different, Δ is a relatively large value and this coupling process has a serious phase mismatch. Using the rotating wave approximation, we perform an approximation as follows:

$$\kappa^2 \ll \Delta^2 \tag{7}$$

this approximation is reasonable as $\mathrm{Q_i}$ is quite sensitive to the loss of fundamental mode, even a small amount of inter-mode coupling can severely deteriorate the Q of the MRR. Therefore, the amplitude of the two modes could be rewritten as follow:

$$A(z) \approx 1 \tag{8}$$

$$B(z) \approx -j\frac{\kappa}{\beta_c}\sin(\beta_c z)\, e^{+j\Delta z} \tag{9}$$

through the RWA, we can obtain $\beta_c \approx \Delta$. In this way, the amplitude of the $\mathrm{TE_0}$ is considered almost invariant. The phase term in equation.(9) indicates that $B(z)$ is simultaneously affected by the propagation constants of both modes. Considering that in the proposed partial EB (the combination of Euler bend and arc bend), the coupling properties of the two bends are different, we utilized the piecewise function to analyze it separately:

$$E_0(z) = \mathrm{C}_0 e^{-j\beta_0(z)z} \tag{10}$$

$$E_1(z) = \begin{cases} \frac{\kappa}{\beta_c}\sin(\beta_c z)\, e^{-j\frac{(\beta_1(z)+\beta_0(z))}{2}z - j\frac{\pi}{2}}, & \text{in EB} \\ \mathrm{C}_1 e^{-j\beta_1(z)z}, & \text{in arc} \end{cases} \tag{11}$$

$$I \propto (E_1 + E_0) * (E_1^* + E_0^*) \tag{12}$$

where C_0 and C_1 represent the constants for amplitude. I represent the intensity pattern. In equation.(10), the TE_0 is approximated as unaffected by the inter-mode coupling, and the variable β_0 is dependent on the changing curvature along the bent waveguide. It can be explained that although a small amount of TE_0 is gradually coupled into TE_1 in Euler bend, this part accounts for a small proportion of the total. As for TE_1 in an arc waveguide, the fixed curvature maintains the orthogonality in the silicon photonics (SiPh) strip waveguide so we consider it without inter-mode coupling. In equation.(11) for EB, the sinusoidal function in magnitude term indicates the power ratio of TE_1 is zero at the input port and gradually increases, which accurately reflects the physical behavior of light propagation from the straight waveguide into the bent waveguide. It is worth mentioning that although we successfully obtain the analytic solution, the calculation of the amplitude term could be omitted since it requires complex numerical calculations. In fact, our objective is to determine the position of the reciprocal point rather than its intensity. Consequently, we place more emphasis on the phase information between the two modes, which can be translated into intensity information via Equation.(12) to determine the position, rather than a complete electromagnetic field solution.

Supplementary note II: Proof of using time-reversal symmetry to regulate spatial mode interaction within a bent waveguide

Here we make a detailed formula derivation. Since the 180° bent waveguide is a highly geometrically symmetrical structure, as shown in Fig.1.d and f, the structure from "Mid" to "Out" is the same as from "Mid" to "In", which means they can be summarized as the same scattering matrix $\mathbf{S}$. Assumed that the field at Mid is denoted by E_m. When the reciprocal point is located at the "Mid", $\boldsymbol{E_m}$ can be expressed as:

$$\boldsymbol{E_m} = e^{j\phi} \begin{pmatrix} A \\ \pm B \end{pmatrix} \tag{13}$$

Where A and B represent the amplitude of the TE_0 and TE_1 in the middle of the bend, respectively. $\pm$ represents that the reciprocal point is a maximum or a minimum (The phase relationship of TE_0 and TE_1 is in-phase or anti-phase). ϕ represents the phase shift. Now we analyze the time reversal path, i.e. from "Mid" to "In". Since only TE_0 is launched at "In", the following expression is always established according to the time-reversal symmetry:

$$\mathbf{S} \cdot \boldsymbol{E_m^*} = e^{-j\phi} \mathbf{S} \cdot \begin{pmatrix} A \\ \pm B \end{pmatrix} \equiv \begin{pmatrix} 1 \\ 0 \end{pmatrix} \tag{14}$$

Where $\boldsymbol{E_m^*}$ is the conjugate of $\boldsymbol{E_m}$, representing the time reversal state of $\boldsymbol{E_m}$. Next, we analyze the evolution of $\boldsymbol{E_m}$ from "Mid" to "Out". The expression is as follows:

$$\mathbf{S} \cdot \boldsymbol{E_m} = e^{j\phi} \mathbf{S} \cdot \begin{pmatrix} A \\ \pm B \end{pmatrix} = e^{2j\phi} \cdot e^{-j\phi} \mathbf{S} \cdot \begin{pmatrix} A \\ \pm B \end{pmatrix} = e^{2j\phi} \begin{pmatrix} 1 \\ 0 \end{pmatrix} \tag{15}$$

We can see from the expression that at this time we obtain the pure TE_0 at "Out". On the other hand, when the reciprocal point is not located at the center of the bend, $\boldsymbol{E_m}$ can be expressed as:

$$\boldsymbol{E_m} = e^{j\phi} \begin{pmatrix} A \\ e^{j\theta} B \end{pmatrix} \tag{16}$$

Where θ represents the phase difference between two modes. At this time, the time-reversal symmetry still works. Equation. (14) is now rewritten as:

$$\mathbf{S} \cdot \boldsymbol{E}_{\boldsymbol{m}}^{*} = e^{-j\phi}\mathbf{S} \cdot \begin{pmatrix} A \\ e^{-j\theta}B \end{pmatrix} \equiv \begin{pmatrix} 1 \\ 0 \end{pmatrix} \tag{17}$$

However, now equation. (15) does not work, since now the expression is as follows:

$$\mathbf{S} \cdot \boldsymbol{E}_{\boldsymbol{m}} = e^{j\phi}\mathbf{S} \cdot \begin{pmatrix} A \\ e^{j\theta}B \end{pmatrix} = e^{2j\phi} \cdot e^{-j\phi}\mathbf{S} \cdot \begin{pmatrix} A \\ e^{j\theta}B \end{pmatrix} \neq e^{2j\phi} \begin{pmatrix} 1 \\ 0 \end{pmatrix} \tag{18}$$

Therefore, by controlling the position of the reciprocal point, according to the time-reversal symmetry, we can obtain the different coupling situations of the two spatial modes in the bent waveguide.

Supplementary note III: Details about the bend structures

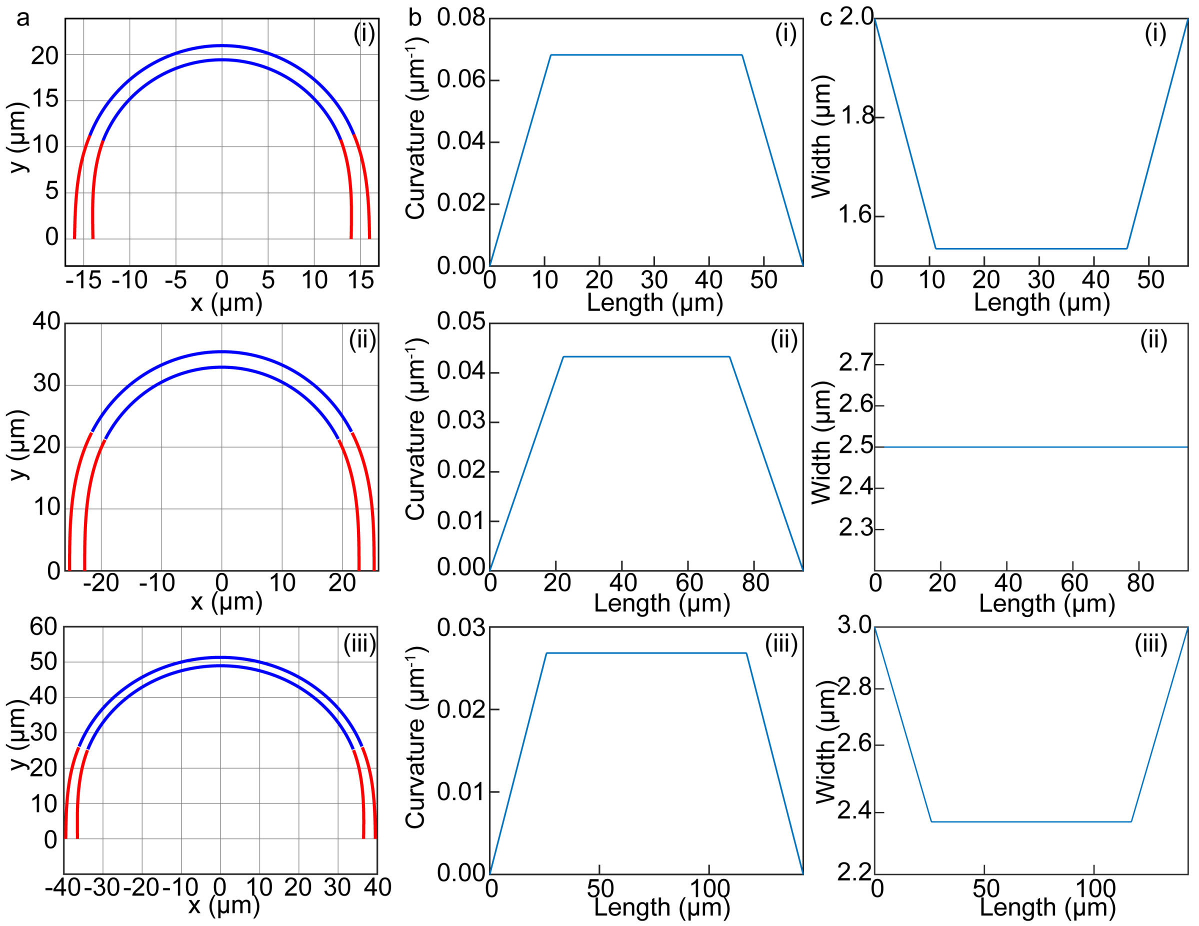

Fig. S2: Details for the bend structures shown in the main text. **a**, The geometry of three bends. Blue: arc bend. Red: Euler bend. **b** and **c**, The variations in curvature and width in three 180° bend.

As shown in Fig.S2 (a), the proposed bend structures are composed of Euler bend and arc bend. The Euler bend can be expressed as:

$$x(s) = x_0 + \int_0^s \cos\left(\frac{1}{2}\kappa'\tau^2\right) \mathrm{d}\tau \tag{19}$$

$$y(s) = y_0 + \int_0^s \sin\left(\frac{1}{2}\kappa'\tau^2\right) \mathrm{d}\tau \tag{20}$$

Where κ' is the curvature change rate. s is the curvilinear abscissa. In Euler bend, our design scheme is to make the width of the waveguide change linearly with the linear change of curvature. Here the width changes of the three bends are from 2 μm to 1.535 μm, from 2.5 μm to 2.5 μm and from 3 μm to 2.39 μm, respectively. Note that variations in width

are not strictly necessary since it is just aimed to provide a new degree of design freedom, and there is more than one way to change the width. The minimum radii of the three bends (the radius of the arc) are 14.6484 μm, 23.1113 μm and 37.2538 μm, respectively.

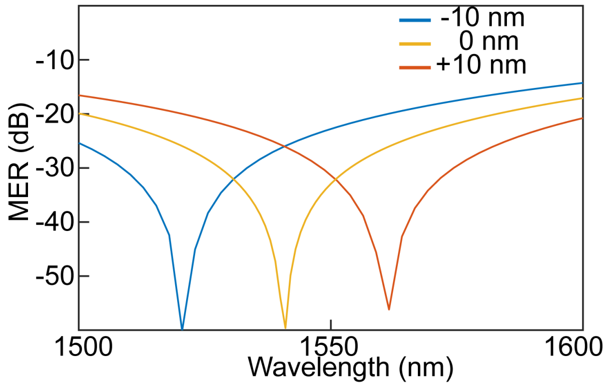

Fig. S3: The simulation result of the optical bandwidth with fabrication error

Since we use the first bend to construct the rMRR, here we show the mode extinction ratio (MER) in the optical bandwidth from 1500 nm to 1600 nm in Fig.S3. The optical bandwidth with MER below -30 dB is around 24 nm. We also simulated the fabrication error of the waveguide within the range of ±10 nm. From the simulation results, it can be seen that the low MER range is still above 20 nm. We can also adjust the center wavelength of the low MER range to be near 1550 nm again utilizing microheaters. In the simulation, we increased the refractive index of the silicon material by 0.01 to represent changes in temperature with a fabrication error of -10 nm. As displayed in Fig.S4, this adjustment resulted in the position of the lowest MER point returning to near 1550 nm.

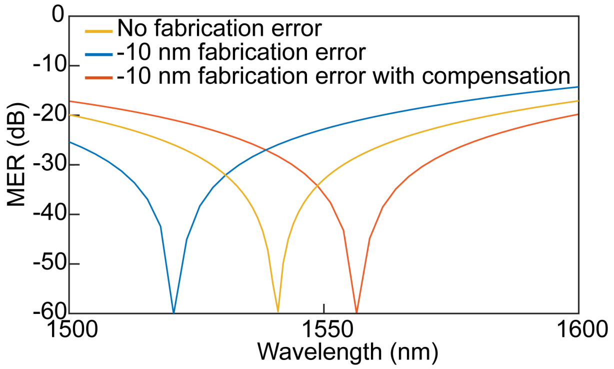

Fig. S4: The compensation for the fabrication of -10 nm. The refractive index of the silicon material is increased by 0.01. Yellow curve: without fabrication error. Blue curve: with fabrication error of -10 nm. Red curve: compensation through thermo-optic effect under a fabrication error of -10 nm.

Supplementary note IV: Details for using the modified CMT to calculate the bend structure

To analyze the proposed bend by the modified coupled mode equations, we need to determine the propagation constants β_0 and β_1 of TE_0 and TE_1 at different widths and curvatures, which can be achieved by calculating the mode distribution in the cross-section of the waveguide. We fix the width of the waveguide and calculate its mode field distribution and effective refractive index at different curvatures from 0 to 0.1 μm^{-1}. Then, we changed the width of the waveguide and did the same, and finally calculated the width of the waveguide from 3 μm to 1.5 μm. The calculated took-up table can be found in Fig.S5. Therefore, once the look-up table is established, the coupling process of two spatial modes in any structure can be quickly analyzed. We can divide the specific bend into a number of small segments. Each segment is considered an arc bend with constant curvature and width. The eigenmodes of TE_0 and TE_1 in the bend can be determined and the corresponding effective refractive index can be obtained. After calculating the phase accumulation of each segment and integrating them, we can know the phase of the two modes TE_0 and TE_1 propagating in the bend. Through equation (12), the position of the reciprocal point can be obtained.

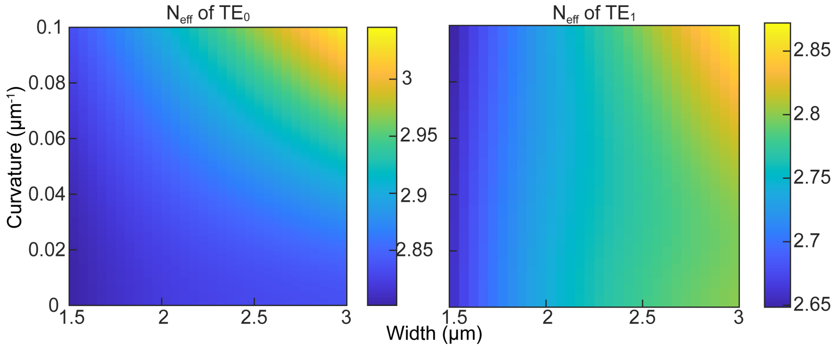

Fig. S5: Calculated look-up table for the effective refractive index of TE_0 and TE_1

Supplementary note V: Thermo-optic effect on the change of electromagnetic field distribution in the bent waveguide.

The proposed bending region is a combination of Euler bend and arc bend. In an arc bend, both the curvature and width of the waveguide remain constant. Consequently, under this structure, the propagation of this mode can be regarded as quasi adiabatic with almost no inter-mode crosstalk. The Euler bend has a larger curvature change rate compared with the conventional one, as shown in Fig.1.b in the manuscript. It is nonadiabatic evolution and can result in the power conversion from TE_0 to TE_1. Therefore, we can observe clear beating or interference patterns along the propagation of the fields and we introduce the concept of reciprocal point (RP). As the nature of interference, such electromagnetic field intensity distribution (the position of RP) is dependent on the relative phase between two modes, which can be modified by the thermo-optics effect via the micro-heaters. The position of the RP determined the equivalent power conversion from TE_0 to TE_1. When the RP is situated in the middle of the bend, the time-reversal symmetry method enables us to infer that the intensity of TE_1 at the output port is currently zero. As the RP deviates from the middle of the bend, the proportion of TE_1 incrementally increases with the offset of the RP. This method is convenient to design the desired device with specified functionality. In order to better observe the electromagnetic field distribution, we displayed more 3D FDTD simulation results (corresponding to the results in Fig.S9.a) to analyze the position changes of the RP and the TE_1 proportion at the output when the temperature changes (by changing the refractive index of silicon), as shown in the enlarged view in Fig.S6.b. As the RP deviates from the middle of the bend, the TE_1 proportion at the output port incrementally increases with the offset of the RP (these calculated proportions at the output port is displayed in Fig.S9.a).

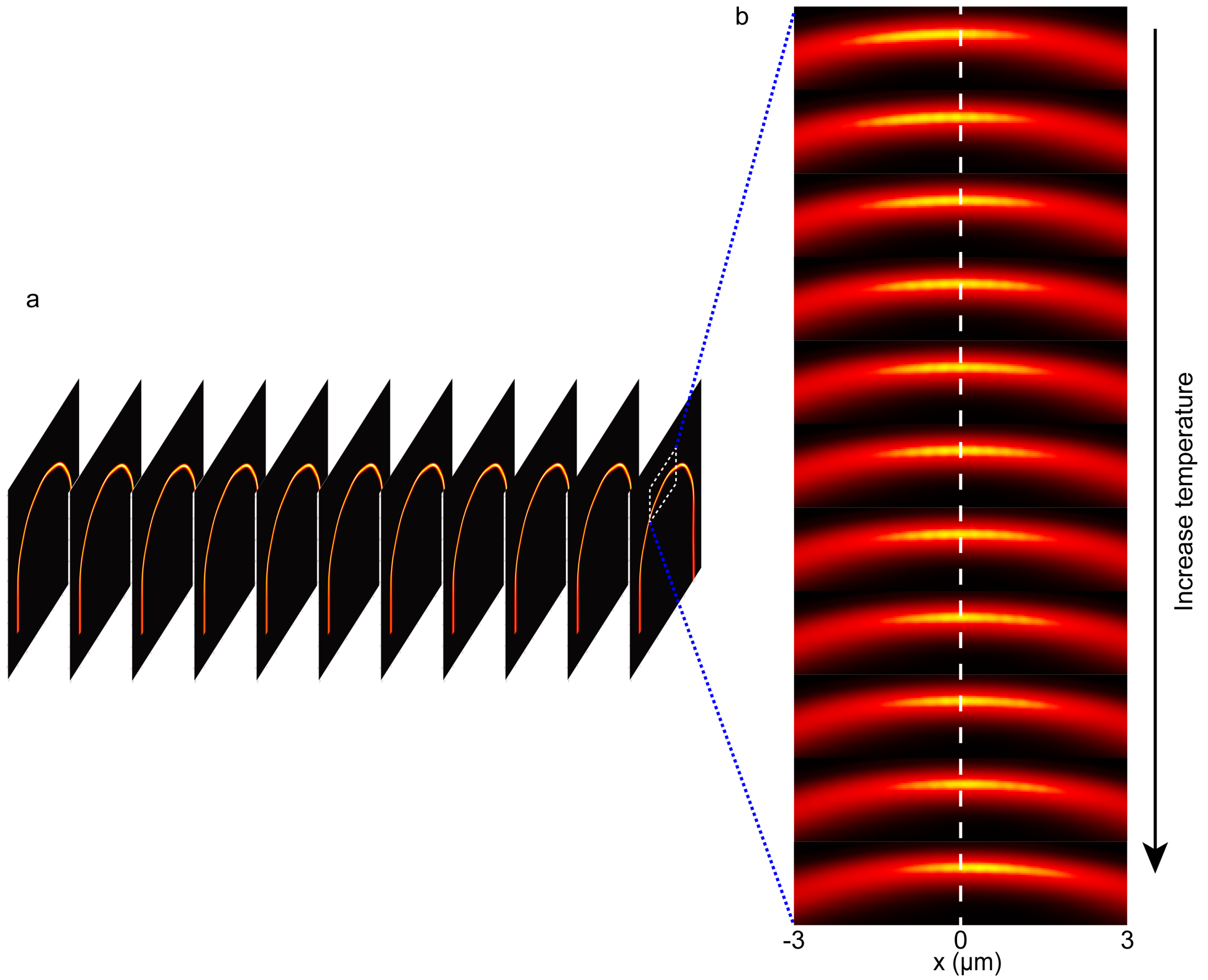

Fig. S6: **a**, the 3D FDTD simulations result under various temperatures (with the refractive index of silicon increasing from left to right). **b**, the enlarged view of **a**. The position of RP is moved with the increasing of temperature.

Supplementary note VI: The details of other resonances peaks in Fig.2

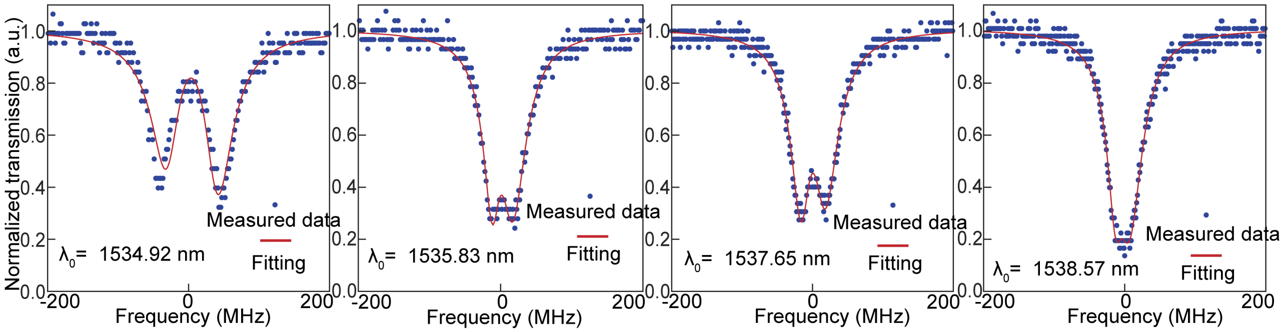

Fig. S7: The enlarged view of four resonance peaks.

Here, we present other resonance peaks from Fig.2.e that exhibit mode splitting due to backscattering. Through fitting[1], the frequency linewidths are 47.55 MHz, 35.42 MHz, 37.42 MHz and 34.35 MHz, as shown in Fig.S7.

Supplementary note VII: The details of filter's passbands at different frequency bands

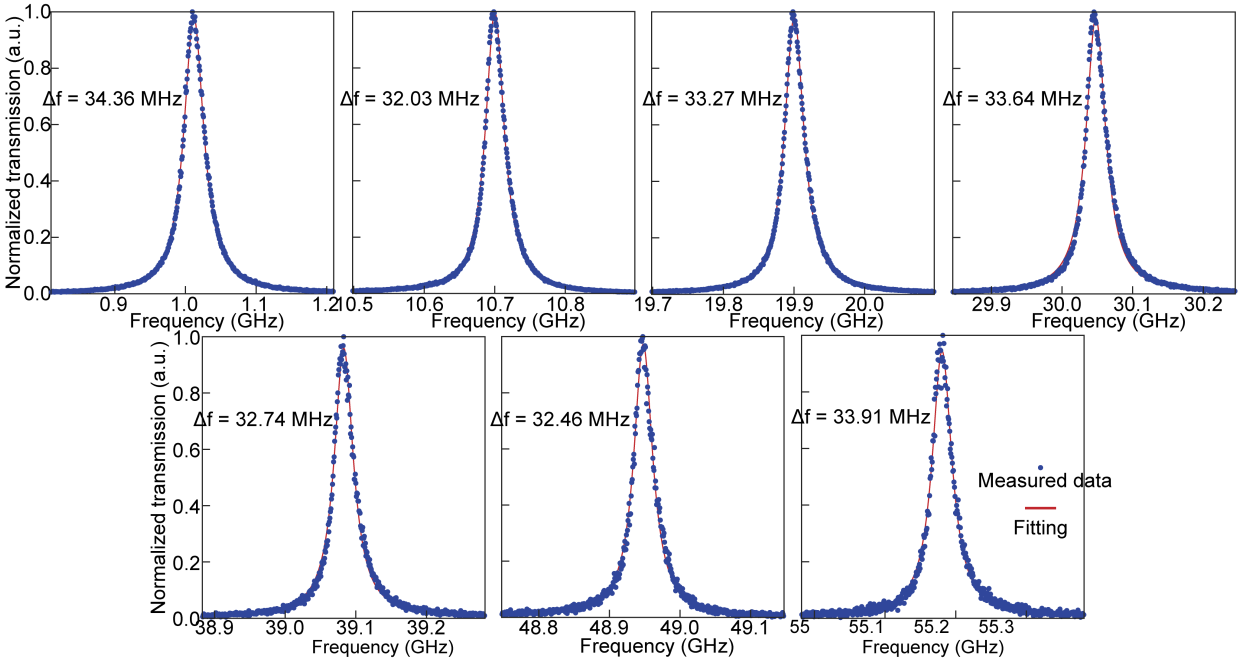

Fig. S8: The enlarged view of the filter's passbands at different frequency bands.

Fig.S8 shows the view of the filter's passbands at different frequency bands with a frequency sweeping range of 0.4 GHz. The 3-dB bandwidths of the passbands are all approximately between 32 MHz to 34 MHz, demonstrating good consistency.

Supplementary note VIII: The spectral bifurcation physical properties in the photonic molecule with the manipulation of thermo-optic effect

The concept of photonic molecule is analogue to the hydrogen molecule [2]. The electronic energy level of two separated hydrogen atom should be the same. When the two atoms get close, the inter-atom coupling process will make the origin degenerated energy level undegenerated. Similarly, the microring resonator can be viewed as a hydrogen atom and the resonant frequency can be analogue to the energy level. Under the coupling between two resonators, the degenerated or same resonant frequency will split into two different resonant frequencies. The coupling rate is the most important coefficient for the control of photonic molecule. The split frequency or the frequency difference of split resonances is determined by the coupling rate between the two resonators.

Here, instead of using two resonators, we use a single resonator and the photonic molecule is formed by resonances of two different transverse modes. Inter-mode coupling is induced and controlled in the 180° curved waveguides. As shown in Fig.2.a in the manuscript, we displayed three types of 180° curved waveguides. We can observe clear beating or interference pattern along the propagation of the fields, indicating the existence of multimode due to inter-mode coupling. As the nature of interference, such electromagnetic field intensity distribution is dependent on the relative phase between two modes. The relative phase can be tuned by changing the refractive index of the material even in the same bend structure via thermal-optics effect. Only when the reciprocal point (RP) is at the center, or in other word, the interference pattern in the bend is symmetry, the output will be pure TE_0 mode, indicating minimal coupling rate between the TE_0 and TE_1. While as the interference pattern in the bend is asymmetry, part of the input TE_0 mode will be converted into TE_1 mode, indicating the effective coupling between the TE_0 and TE_1 mode after passing the 180° bend.

For a clear explanation, we can use the relative phase difference $\Delta\varphi$ between the TE_0 and TE_1 at the center of the waveguide to value the degree of symmetry and asymmetry. And we get the effective inter-mode coupling rate after passing the 180° bend under different $\Delta\varphi$ as shown in Fig.S9 a. Due to the changing of the coupling rate μ_t, the photonic molecule will feature different spectral bifurcation physical properties. Here, we employ the coupling

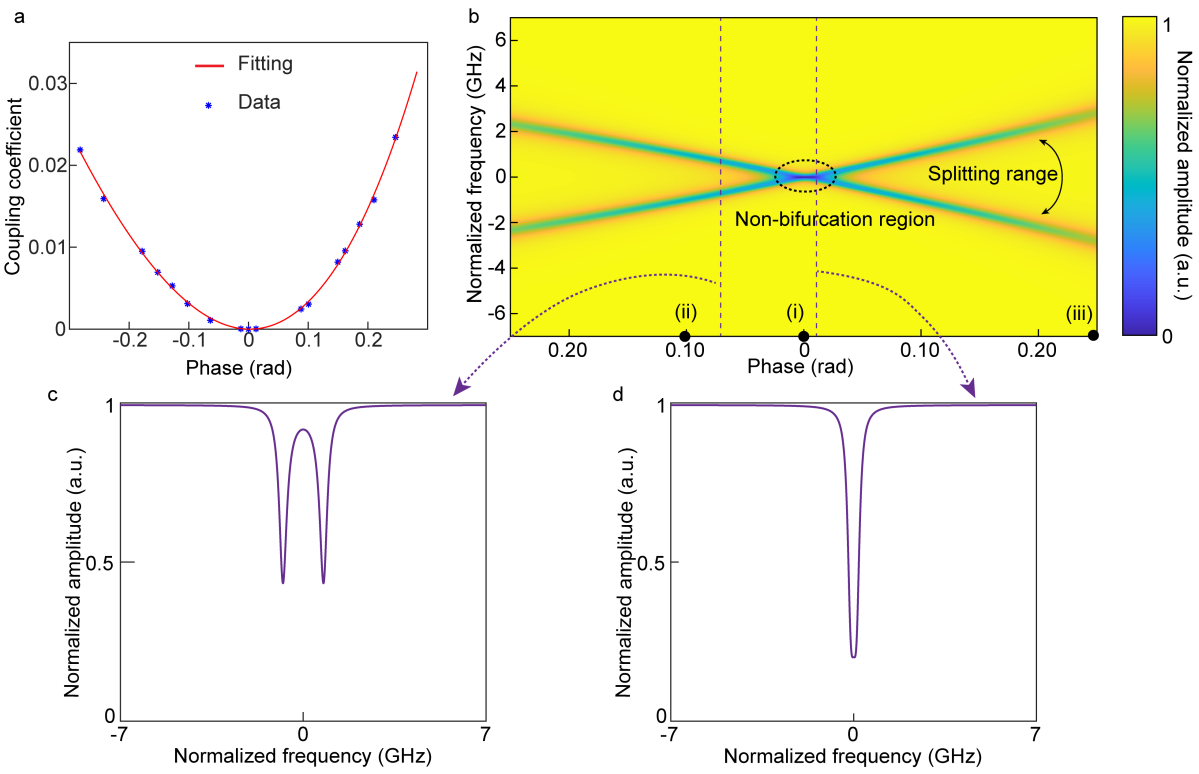

Fig. S9: **a**, the relationship between the relative phase difference and coupling rate. **b**, the relationship between the phase difference of two spatial modes when they are in the middle and the spectral bifurcation physical properties. **c,d** obvious and not obvious frequency bifurcation physical properties.

mode equations to calculate the field in the single microring:

$$\frac{\partial E_0}{\partial t} = \left(-j\delta_0 - \frac{1}{\tau_0}\right) E_0 + j\mu_t E_1 + \mu_0 E_0 \tag{21}$$

$$\frac{\partial E_1}{\partial t} = \left(-j\delta_1 - \frac{1}{\tau_1}\right) E_1 + j\mu_t E_0 + \mu_1 E_1 \tag{22}$$

Where E_0 and E_1 are the intracavity fields for TE_0 mode and TE_1 mode. τ_0 and τ_1 are the photon lifetimes of TE_0 mode and TE_1 mode. $\delta_0 = \omega - \omega_0$ and $\delta_1 = \omega - \omega_1$ are the frequency detunings, where ω_0 and ω_1 are the resonant frequencies. Here we set $\omega_0 = \omega_1$. We solve Eq. (21,22) under different μ_t due to the change of $\Delta\varphi$. The transmission spectra are shown in Fig.S9.b and shows obvious bifurcation physical properties with the change of $\Delta\varphi$. At first, there is no mode splitting since $\Delta\varphi = 0$, corresponding to the minimal coupling rate. As the $\Delta\varphi$ is quite small, there is no obvious splitting (Fig.S9.d). It is because the slight mode splitting is sheltered by the linewidth of the resonant. In other words, now the

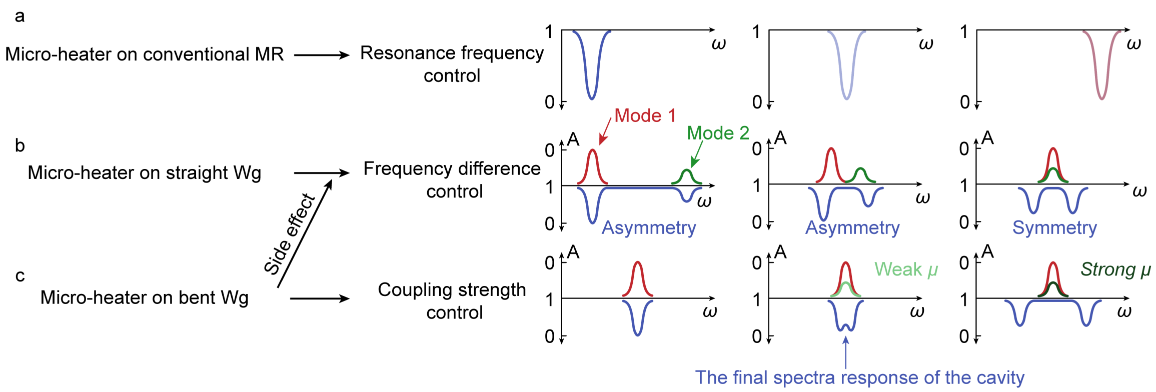

Fig. S10: The operation schematic of the spectra response of the photonic molecule based on the cavity via micro-heaters. MR: microring; μ_t: couping rate.

mutual coupling rate is lower than the field decay rates (δ_0). Then, as the phase difference continues to increase, the frequency bifurcation physical properties emerge (Fig.S9.c). The large mutual coupling rate can correspond to the higher frequency doublet gap.

In order to realize the above-mentioned splitting control function of photonic molecules, we need two types of micro-heaters as shown in Fig.S10 and also in Fig.4.b in the main text. In the conventional microring (MR), the micro-heaters have the same effect wherever it is placed on the MR, that is changing the propagation constants of the mode existing in the MR. Since silicon is a material with a positive thermo-optical coefficient, when the micro-heater is powered, the propagation constant of the mode increases, resulting in the red shift of the resonance peak of the MR in the frequency domain, as shown in Fig.S10.a. Many works have taken advantage of this feature to move the center frequency of the resonant peak. In our work, however, the thermal-optics effect features more complicated functionalities. The micro-heaters placed on the straight waveguide serve to alter the difference in propagation constants between the TE_0 and TE_1 modes, as shown in Fig.S10.b. This occurs due to the distinct responses of these modes to the thermal field, which is attributable to their different mode field distributions. According to Eq.(21-22), the functionality of this type of micro-heater is to change the frequency difference of resonant frequency between TE_0 and TE_1, that is $\delta_0 = \omega_1 - \omega_0$. When $\delta_0 = 0$, it corresponds to the symmetric mode splitting in the frequency domain as shown in Fig.4.d in the manuscript. When $\delta_0 \neq 0$, it represents the asymmetric mode splitting, which means the resonance peaks from TE_0 and TE_1 are not aligned in the frequency domain. As for the micro-heaters placed on the bent waveguide of

the proposed MR, the functionality is to modify the mutual coupling coefficient between TE_0 and TE_1 mode, as shown in Fig.S10.c. We have explained this coupling mechanism above and it is related to the relative phase difference between two modes. By changing the voltage of this type of micro-heater, the refractive index in bent region can be modified to move the position of RP, corresponding to equivalently change the abscissa value of Fig.S9.b, so that the mode splitting can be effectively adjusted because of the change of coupling rate. Also, it has the side effect of changing the propagation constants of TE_0 and TE_1. Therefore, the micro-heater on the straight waveguide have extra assignments to compensate for it and correct the mode splitting from asymmetry to symmetry in the frequency domain.

Supplementary note IX: Simulation results for other material platform

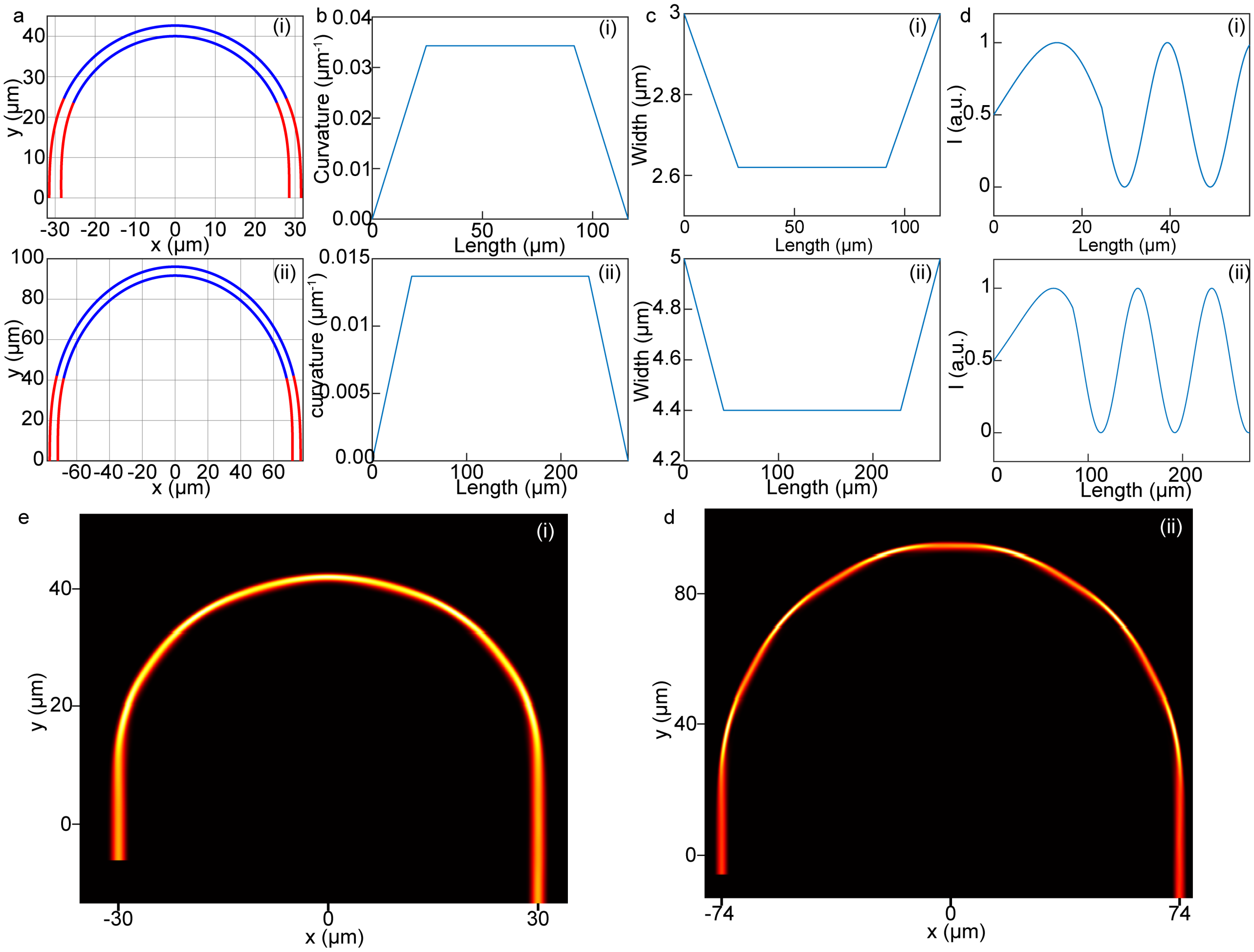

Fig. S11: Details for the bend structures in SiN material platform a, the geometry of two bends. **b** and **c** The variations in curvature and width. **d,** the calculated intensity pattern by the modified coupled mode equations.

To demonstrate the proposed spatial mode control method is solid in other material platforms, we use silicon nitride (SiN) material to construct the waveguide and carry out the calculation. The thickness of the waveguide is 800 nm, covered with SiO_2 cladding. The details of the geometry, curvature change, width change, the position of the reciprocal point using modified mode theory calculations and FDTD simulations are shown in Fig.S11. We can see that in SiN material platform, the reciprocal point still exists. The chord lengths of the bends are 60 μm and 148 μm, respectively. The width changes of the two bends are from 3 μm to 2.62 μm, and from 5 μm to 4.4 μm, respectively. The minimum radii of the three bends (the radius of the arc) are 29.1470 μm and 73.0035 μm, respectively. And the

modified coupled mode theory is well-matched with the FDTD results. The lookup table for curvature, width, and effective refractive index relationship for the 800 nm thick SiN waveguide is shown in Fig.S12.

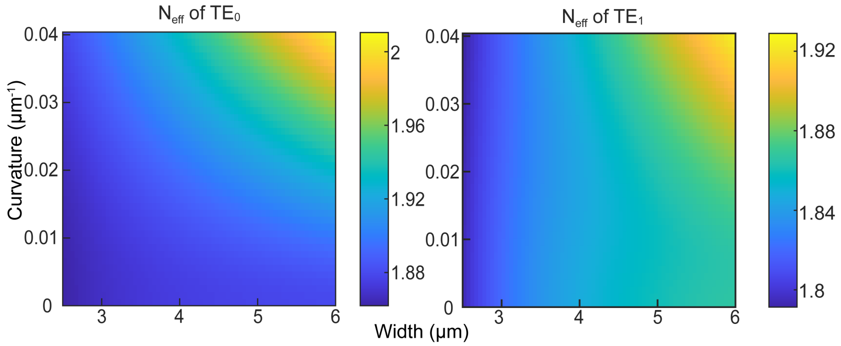

Fig. S12: Calculated look-up table for the effective refractive index of TE_0 **and** TE_1 **in SiN platform**

---

[1] Li, A., Van Vaerenbergh, T., De Heyn, P., Bienstman, P. & Bogaerts, W. Backscattering in silicon microring resonators: a quantitative analysis. *Laser & Photonics Reviews* **10**, 420–431 (2016).

[2] Rakovich, Y. P. & Donegan, J. F. Photonic atoms and molecules. *Laser & Photonics Reviews* **4**, 179–191 (2010).
